# CHANGES IN COGNITION ARE RELATED TO PERSONALITY CHANGES IN MIDDLE AND LATER ADULTHOOD

**DOI:** 10.1093/geroni/igz038.583

**Published:** 2019-11-08

**Authors:** Yujun Liu, Margie E Lachman

**Affiliations:** 1 Brandeis University, Waltham, United States; 2 Brandeis University, Waltham, Massachusetts, United States

## Abstract

Previous studies have identified an association between personality and cognition in later life. Those with more stable personalities have better cognitive functioning. And those with more cognitive decline have higher neuroticism. We examined the association between changes in both personality and cognition in the same model, which has not been systematically investigated. Data were from the Midlife in the United States (MIDUS) study, a national survey that included 4268 participants ages 35 to 85 at the second wave. The Big Five Personality traits and cognitive function variables (episodic memory and executive functioning) were from waves two and three. The analysis included a latent change score model and a cross-lagged panel design using Mplus. The results show that personality changes and cognitive changes over 9 years are correlated. Cross-lagged findings indicate that cognitive functioning is positively related to changes in conscientiousness, agreeableness, openness, and extraversion and negatively related to changes in neuroticism. The findings advance our understanding of the association between changes in personality and cognition. The impact of cognitive change on personality stability and the role of personality traits for maintaining cognitive function in later life are discussed. The results have implications for developing interventions to maintain or enhance cognitive functioning and personality in middle and later adulthood.

